# A novel method for automated crystal visualization and quantification in murine folic acid-induced acute kidney injury

**DOI:** 10.1152/ajprenal.00140.2023

**Published:** 2023-10-26

**Authors:** Ahmad Kamal Hamid, Eva Maria Pastor Arroyo, Sung Sik Lee, Carsten Alexander Wagner, Daniela Egli-Spichtig

**Affiliations:** ^1^Institute of Physiology, University of Zurich, Zurich, Switzerland; ^2^Swiss National Centre of Competence in Research NCCR Kidney.CH, Zurich, Switzerland; ^3^Scientific Center for Optical and Electron Microscopy, ETH Zurich, Zurich, Switzerland; ^4^Institute of Biochemistry, Department of Biology, ETH Zurich, Zurich, Switzerland

**Keywords:** acute kidney injury, fibroblast growth factor 23, folic acid crystal visualization, quantitative image analysis, sex differences

## Abstract

Folic acid (FA)-induced acute kidney injury (FA-AKI) is an increasingly prevalent rodent disease model involving the injection of a high dose of FA that culminates in renal FA crystal deposition and injury. However, the literature characterizing the FA-AKI model is sparse and dated in part due to the absence of a well-described methodology for the visualization and quantification of renal FA crystals. Using widely available materials and tools, we developed a straightforward and crystal-preserving histological protocol that can be coupled with automated imaging for renal FA crystal visualization and generated an automated macro for downstream crystal content quantification. The applicability of the method was demonstrated by characterizing the model in male and female C57BL6/JRj mice after 3 and 30 h of FA treatment. Kidneys from both sexes and timepoints showed a bimodal distribution of FA crystal deposition in the cortical and medullary regions while, compared with males, females exhibited higher renal FA crystal content at the 30-h timepoint accompanied by greater kidney weight and higher plasma urea. Despite comparable plasma phosphate concentrations, FA-AKI resulted in a substantially more elevated plasma intact fibroblast growth factor 23 (FGF23) in females, reflected by a similar pattern in osseous *Fgf23* mRNA expression. Therefore, the presented method constitutes a valuable tool for the quantification of renal FA crystals, which can aid the mechanistic characterization of the FA-AKI model and serves as a means to control for confounding changes in FA crystallization when using the model for investigating early and prophylactic AKI therapeutic interventions.

**NEW & NOTEWORTHY** Here, we describe a novel method for the visualization and quantification of renal folic acid (FA) crystals in the rodent FA-induced acute kidney injury (FA-AKI) model. The protocol involves a straightforward histological approach followed by fully automated imaging and quantification steps. Applicability was confirmed by showing that the FA-AKI model is sex-dependent. The method can serve as a tool to aid in characterizing FA-AKI and to control for studies investigating prophylactic therapeutic avenues using FA-AKI.

## Graphical abstract

**Figure:**
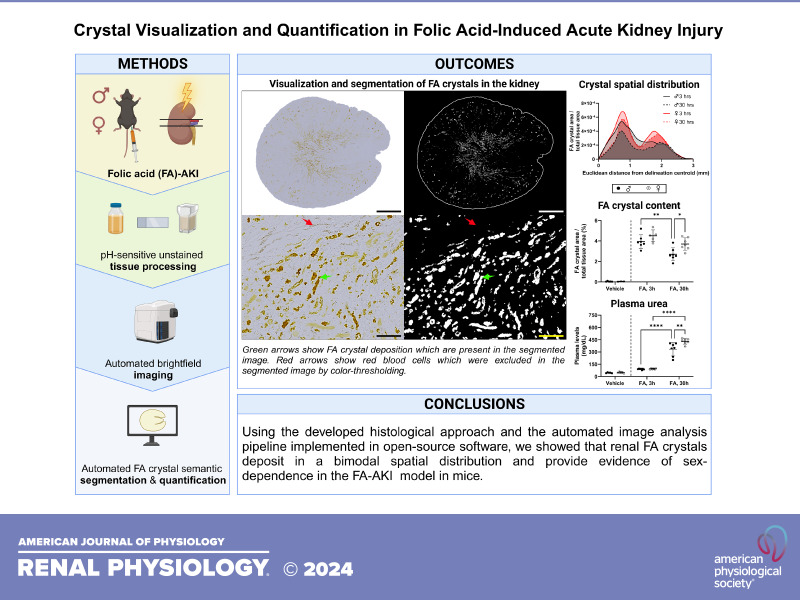

## INTRODUCTION

Acute kidney injury (AKI) is a common medical emergency characterized by a sudden increase in serum creatinine or the presence of oliguria precipitated by a pre-, intra-, or postrenal insult or dysfunction (1). Kidney dysfunction causes an accumulation of metabolic byproducts and a dysregulation in electrolyte homeostasis (2). For example, experimental AKI is typically accompanied by a dramatic rise in plasma phosphate (P_i_) and fibroblast growth factor 23 (FGF23), a bone-derived phosphaturic hormone with a well-established proclivity for extra-osseous expression under pathological conditions (3–5). Plasma P_i_ and FGF23 are independently associated with higher renal disease severity and worse prognosis (6–9).

An increasingly prevalent disease model to study AKI is folic acid (FA)-induced AKI (FA-AKI). An initial report for xanthopterin, a precursor of FA, to induce a transient enlargement of rat kidneys prompted further investigations of the presumed hypertrophic properties of pteridines, culminating in revealing that a high-dose FA (pteroylglutamic acid) injection can lead to crystal deposition in renal tubules (10–13). Subsequently, FA was shown to produce tubular lesions, reduce glomerular filtration rate (GFR), and dysregulate sodium and water balance, recapitulating the hallmarks of clinical AKI and enabling the proposal of high-dose FA as an experimental model in rats and later in mice (14–16). FA-AKI is currently widely used on account of its short induction-to-onset duration, facile implementation, high symptomatic reproducibility, and cost effectiveness. As opposed to surgical, endotoxin-, and nephrotoxin-induced models, FA-AKI is noninvasive, requires minimal training, has no reported extrarenal toxicity, and is environmentally innocuous.

The literature characterizing FA-AKI is sparse and outdated, as the model’s utility superseded the priority for its elucidation. Although FA-AKI has been repeatedly demonstrated in both male and female mice, studies reporting on sex differences are extremely limited (4, 17–19). Moreover, while some studies reported renal FA crystal deposition in the distal nephron—cortical distal convoluted tubules; cortical, medullary, and papillary collecting ducts; and outer medullary loops of Henle—others reported that the site of injury also extends to the proximal tubule (20–24). A method for the visualization and quantification of FA crystals is requisite for such characterization studies. Although renal FA crystal deposition was demonstrated previously by way of light and electron microscopy, the approaches were qualitative and unaccompanied by a detailed methodology for tissue fixation and sample preparation that would preclude crystal elution (12, 14, 22, 25).

In this work, we established a simple tissue fixation and sample preparation protocol that preserves deposited renal FA crystals in mice with FA-AKI for automated bright-field microscopy and devised a fully automated macro for the quantification of whole slice crystal content as well as their spatial distribution using the open-source software FIJI. To demonstrate the utility and robustness of the method, we studied crystal clearing over time in FA-AKI and whether the model bears sex dependence in terms of crystal deposition and renal injury and dysfunction. This study offers a method for the quantification of renal FA crystals which could be of help in the mechanistic characterization of the FA-AKI model in future studies and serve as a tool to control for confounding changes in FA crystallization when using the model for investigating early and prophylactic AKI therapeutic interventions.

## METHODS

### Animals and FA-AKI Induction

Male and female C57BL6/JRj mice were purchased from Janvier Labs and allowed to acclimate for 1 wk in a conventional animal facility at the Laboratory Animal Services Center, University of Zurich. Animal housing conditions included a 12:12-h light-dark cycle, an ambient temperature of 22 ± 2°C and ad libitum access to chow and water. The chow contained 0.6% wt/wt phosphorous as calcium or sodium salts, 1% wt/wt calcium, 3,500 IU/kg cholecalciferol, and 16 mg/kg FA (S9151-E714, ssniff Spezialdiäten). All procedures conducted throughout this work were in accordance with the Swiss animal welfare laws and guidelines for animal care and approved by the Zurich Veterinary Office under license number 169/2019.

FA-AKI was induced with a single intraperitoneal injection of 250 mg/kg FA (F7876, Sigma) thoroughly dissolved at a concentration of 25 µg/µL in 150 mM NaHCO_3_ at pH 7.4 or vehicle as previously described (5). After 3 or 30 h, spot urine was collected, and the mice were euthanized by exsanguination under continuous inhalant anesthesia (Isoflurane, Piramal Critical Care). Furthermore, urine was collected by bladder puncture and blood was collected by cardiac puncture with a heparinized needle (heparin-sodium, 5 μL, B. Braun). Blood was centrifuged at 6,000 *g* and 4°C for 10 min for plasma separation. Decapsulated kidneys were weighed and normalized to body weight (BW) measured before euthanasia. For histological analysis, a 2-mm mid-transverse section was excised from the kidney.

### Biochemical and Immunoassays

Plasma urea (2020-430, STANBIO), plasma and urinary creatinine (0430-120, STANBIO), and P_i_ (0830-125, STANBIO) were measured using commercial kits. Plasma intact (i)FGF23 was determined by a commercially available ELISA kit (60-6800, Quidel). Plasma FA was measured using an electrochemiluminescence immunoassay (Elecsys Folate III, Roche) by the Institute of Clinical Chemistry, University Hospital Zurich.

### RNA Extraction and Real-Time Semi-Quantitative RT-PCR

Tissues were homogenized using ceramic beads on the Precellys 24 (Bertin Instruments) either in TRIzol reagent (15596026, Invitrogen) followed by chloroform extraction or with NucleoSpin RNA lysis buffer (Macherey-Nagel) for kidney tissue. Total RNA was isolated using the NucleoSpin RNA Mini kit (740955, Macherey-Nagel) as per the manufacturer’s protocol and quantified using the NanoDrop ND-1000 spectrophotometer (Thermo Fisher Scientific). RNA was reverse-transcribed on a thermocycler (SensoQuestGmbH) using the TaqMan Reverse Transcription Reagent Kit (Applied Biosystems): 7.5 ng/μL RNA template, 5.5 mM MgCl_2_, 5 ng/μL random hexamers (79236, Qiagen), 500 μM each dNTP, 0.4 U/mL RNAse inhibitor, 1.25 U/μL multiscribe reverse transcriptase, and RNAse-free water. The PCR thermal profile consisted of 10 min at 25°C, 30 min at 48°C, and 5 min at 95°C.

Real-time semiquantitative PCR (qPCR) was run on either 96- or 384-well plates (4346906 and 4309849, Applied Biosystems, respectively). For probe-based qPCR, the KAPA Probe Fast qPCR kit was used (KK4715, Roche): a 20 µL reaction consisting of 0.1 µM fluorescent probe, 1 µM each of forward and reverse primers, 10 µL Taqman mastermix, 0.4 µL Rox Low passive dye, and 3 µL cDNA template. The PCR thermal profile was composed of 20 s at 95°C, followed by 40 cycles of 1 s at 95°C and 20 s at 60°C. For dye-based reactions, the PowerUp SYBR Green Master Mix was used (A25742, Applied Biosystems): a 20 µL reaction consisting of 1 µM each of forward and reverse primers, 10 µL SYBR Green Master Mix, and 3 µL cDNA template. The PCR thermal profile was composed of 2 min at 50°C and 2 min at 95°C, followed by 40 cycles of 1 s at 95°C and 30 s at 60°C. A melt curve was also conducted for dye-based reactions to confirm specificity. All qPCRs were run on the QuantStudio 6 Pro Real-Time PCR System (Thermo Fisher Scientific). All primer sequences are listed in Table 1.

**Table 1.** Primer and probe sequences used for real-time semi-quantitative PCR

Gene	Oligo	Sequence	Fluorophore/Quencher	Multiplex Compatibility
*Hprt*	Probe	5′-TGC TGA CCT GCT GGA TTA CAT TAA AGC ACT GAA-3′	FAM/BHQ-1, iQ500*	*Gusb, Tbp*
	Forward	5′-CCT CTG TGT GCT CAA GGG G-3′		
	Reverse	5′-GAT CAT TAC AGT AGC TCT TCA GTC TGA-3′		
*Gusb*	Probe	5′-CGA ACC AGT CAC CGC TGA GAG TAA TCG-3′	YYE/BHQ-1	*Hprt, Tbp*
	Forward	5′-CTC ATC TGG AAT TTC GCC GA-3′		
	Reverse	5′-GGC GAG TGA AGA TCC CCT TC-3′		
*Tbp*	Probe	5′CGA ACC AGT CAC CGC TGA GAG TAA TCG--3′	Cy5/BHQ-2	*Hprt, Gusb*
	Forward	5′-GAA ATG CTG AAT ATA ATC CCA AGC GAT-3′		
	Reverse	5′-TTC ACT CTT GGC TCC TGT GC-3′		
*Lcn2*	Forward	5′-TCT GTC CCC ACC GAC CAA T-3′	SYBR green	
	Reverse	5′-GGA AAG ATG GAG TGG CAG ACA-3′		
*Havcr1*	Forward	5′-AGT CAG CAT CTC TAA GCG TGG-3′	SYBR green	
	Reverse	5′-GTC TTC AGC TCG GGA ATG CA-3′		
*Fgf23*	Probe	5′-AGA GGA CGC CGG CTC TGT GGT GAT A-3′	FAM/BHQ-1	
	Forward	5′-TCA GAC CAT CTA CAG TGC CCT-3′		
	Reverse	5′-GGA ACC TTC GAG TCA TGG CT-3′		
*Tnf*	Probe	5′-ATT CGA GTG ACA AGC CTG TAG CCC ACG T-3′	FAM/BHQ-1	*Tgfβ1*
	Forward	5′-CAG ACC CTC ACA CTC AGA TCA TC T-3′		
	Reverse	5′-CCT CCA CTT GGT TTG CT-3′		
*Tgfβ1*	Probe	5′-CCC ATT GCT GTC CCG TGC AGA GCT G-3′	Cy5/BHQ-2	*Tnf*
	Forward	5′-CGT CAG ACA TTC GGG AAG CA-3′		
	Reverse	5′-TGC TAT ATT TCT GGT AGA GTT CCA CAT-3′		
*Il1β*	Probe	5′-TGA GCT TTG TAC AAG GAG AAC CAA GCA AC-3′	FAM/TAMRA	
	Forward	5′-AAT GGA CAG AAT ATC AAC CAA G-3′		
	Reverse	5′-CTG CAG GGT GGG TGT GCC GTC T-3′		
*Il6*	Probe	5′-TGA TGG ATG CTA CCA AAC TGG ATA TAA TC-3′	FAM/TAMRA	
	Forward	5′-AAC AAT CTG AAA CTT CCA GAG ATA C-3′		
	Reverse	5′-GCT ATG GTA CTC CAG AAG ACC AGA-3′		

*iQ500, internal quencher.

Gene expression was quantified using the Livak method, where 2^−ΔCT^ was first calculated for each sample by normalizing to a reference gene, before dividing by the average of all 2^−ΔCT^ values of the vehicle-treated male group to display the fold change. If baseline expression in the vehicle-treated male group was absent, 2^−ΔCT^ values were displayed. For every experiment and tissue, a triplex reaction of reference genes was run (*Hprt*, *Gusb*, and *Tbp*), and the gene that did not significantly vary with treatment or the geometric mean (of 2^−ΔCT^ values) of a combination thereof was used to normalize the results before the fold change calculation (26): kidney (*Tbp*), bone (geometric mean: *Hprt*, *Tbp*), spleen (geometric mean: *Hprt*, *Tbp*), and thymus (*Gusb*).

### Validation of Multiplex qPCRs

Several qPCRs were run in a multiplex fashion (Table 1). To that end, primers and probes were first designed using Primer3web v4.1.0 such that at least one primer or probe spans an exon-exon junction (27–29). Candidate sequences were selected via elimination based on specificity (BLAST), melting temperature proximity (ΔT_m_ < 5°C), and interaction (30). The latter was assessed using the Multiple Primer Analyzer (Thermo Fisher Scientific) and the OligoAnalyzer Tool (Integrated DNA Technologies) based on inter- and intracomplementarity and secondary structure formation, with a threshold Δ*G* of −9 kcal/mol. Multiplex primers and probes (Microsynth) were then validated by generating absolute and relative standard curves for individual uniplex and multiplex reactions. Absolute standard curves were generated using logarithmically diluted synthetic double-stranded DNA (gBlocks, Integrated DNA Technologies) composed of conjoined amplicons separated by five thymine bases. Relative standard curves were generated using logarithmically diluted appropriate tissue cDNA. Multiplex performance was assessed based on comparing both types of standard curves from the uniplex and multiplex reactions of each individual primer and probe set. Differences deemed acceptable were a maximum of 1 C_T_ value difference between the curves at any given point, a correlation coefficient >0.95 for each curve, and an efficiency of 85–110% for both curves with a maximum difference of 5%. Reaction efficiency (*E*) was calculated as per the equation *E* = −1 + 10^−1/slope^.

### Tissue Fixation and Sample Preparation

**Figure 1.**
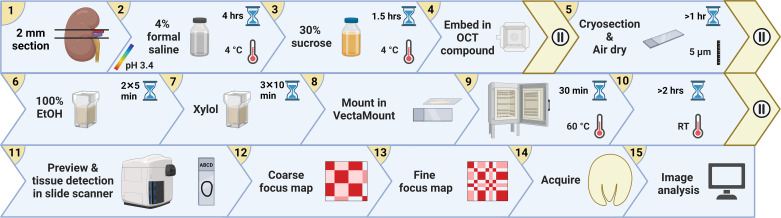
Histological workflow for kidney whole slice imaging after folic acid (FA)-induced acute kidney injury (FA-AKI). To prepare for FA crystal brightfield imaging, a transverse 2-mm mid-section was excised from the kidney and immediately fixed in acidic formal saline, cryoprotected in sucrose, embedded in optimal cutting temperature (OCT) compound, sectioned in a cryostat, air-dried, dehydrated in ethanol (EtOH), cleared in xylol, and mounted in VectaMount. The slides were then previewed in a slide scanner where tissues were manually encircled, and coarse and fine focus maps were then generated before acquisition and image analysis. Pause icons indicate points where the protocol can be interrupted. Created with BioRender.com.

The mid-transverse kidney sections (2 mm) were immediately placed in 4% formal saline and placed on a shaker for 4 h at 4°C: 4% formal saline was prepared by diluting 16% (wt/vol) methanol-free formaldehyde (15710, Electron Microscopy Sciences) in 1.2% (wt/vol) NaCl in distilled deionized water (DDW); final pH of 3.4. The sections were then placed in 30% (wt/vol) sucrose (84097, Sigma) in DDW for 90 min at 4°C on a shaker for cryopreservation, and subsequently embedded in Optimal Cutting Temperature (OCT) compound (4583, Tissue-Tek) and frozen in a liquid nitrogen-cooled liquid-propane bath. Next, 5-µm sections were cut with a cryostat (Leica CM1850) onto glass slides (J1800AMNZ, Epredia), air-dried for a minimum of 30 min (this step is essential for minimizing downstream OCT artifacts), dehydrated in 100% ethanol (20821, VWR) twice for 5 min, cleared in xylol (253-VL54TE, Thommen-Furler) thrice for 10 min, mounted in VectaMount (H-5000, Vector Laboratories), dried in an oven at 60°C for 30 min, and finally air-dried for a minimum of 2 h. Strict adherence to this protocol is paramount, particularly the fixation solution and duration, dehydration/clearing steps, and the mounting medium: optimization experiments indicated that FA crystals can elute within seconds in non-acidic aqueous solutions. The histological workflow is schematically illustrated in Fig. 1.

### Imaging

All slides were imaged in an automated manner using bright-field microscopy with the ZEISS Axio Scan.Z1 slide scanner at the Center for Microscopy and Image Analysis, University of Zurich. Tissue detection was set to manual given the translucence of the unstained tissue: look-up table (LUT) range was adjusted to facilitate visual tissue discernment after preview imaging, and a spline contour was drawn around the sections. After tissue selection, two focus maps were generated using the Hitachi HV-203SCL camera. The coarse-focus map was constructed using the Fluar 5x M27 objective (numerical aperture = 0.25 and working distance = 12.5 mm) with the following parameters: 100% flash intensity; 3-µs exposure; shading correction, auto contrast (tolerance = 19.8), and clipping to valid bits enabled; 14 slices with 30-µm intervals; the center of gravity focus point distribution strategy, and the sharpness measure set to best. The fine-focus map was constructed using the Plan Apochromat 20x M27 objective (numerical aperture = 0.8 and working distance = 0.55 mm) with the following parameters: 87% flash intensity; 4-µs exposure; shading correction, unsharp mask (strength = 1.0, radius = 2, and color mode set to luminance), and clipping to valid bits enabled; 51 slices with 1-µm intervals; the Every *n*^th^ Tile (*n* = 2) focus point distribution strategy; and the sharpness measure set to best. The slides were then imaged with the same parameters as the fine-focus map except for flash intensity (125%) and exposure (3 µs); online stitching was enabled to maximize performance.

### FA Crystal Content Quantification

FA crystal content quantification was conducted using a macro in FIJI software version 1.53t (code deposited and freely available at https://gitfront.io/r/AKMHamid/RWpkdKCDZT4J/FACrystalQuantMacro/). The pipeline involved three fully automated principal steps: *1*) a preprocessing step for the generation of a selection delineating the renal slice, *2*) the quantification of FA crystals using the HSB (hue, saturation, brightness) color space, and *3*) the quantification of total tissue area using the RGB (red, green, blue) color space.

**Figure 2.**
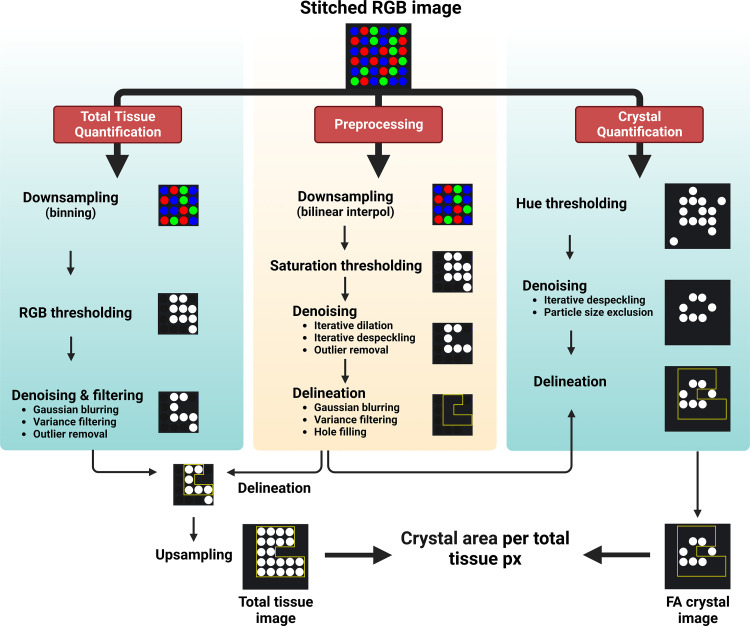
Image analysis pipeline for the quantification of folic acid (FA) crystals in unstained kidney whole slices after FA-induced acute kidney injury (FA-AKI). Image preprocessing involved thresholding in the saturation channel of the hue-saturation-brightness (HSB) color space followed by denoising and filtering to facilitate the delineation of the slice border forming a region of interest (ROI). Total tissue was quantified by thresholding in the red-green-blue (RGB) color space followed by denoising and filtering to render a binary image where a white object (tissue) is punctuated with black islands corresponding to large empty spaces in the tissue. FA crystals were quantified by thresholding in the hue channel of the HSB color space followed by denoising. The previously generated ROI was then applied to the total tissue and crystal binary images to allow for the quantification of the FA crystal pixel count as a percent of the total tissue pixel count within the ROI. Created with BioRender.com.

1) For the preprocessing step, the aim was only to generate a circumscribing selection, thereby allowing for aggressive processing. Images were first downscaled by a factor of 4 using bilinear interpolation with averaging enabled to reduce the computational burden. Each image was then decomposed in the HSB color space, and thresholding of the saturation map was used to isolate tissue from the background. A series of morphological and denoising commands (Dilate, Despeckle, and Remove Outliers) and filters (Gaussian Blur and Variance Filter) were then run to generate a continuous (via Fill Holes command) white object of a perimeter circumscribing the whole renal slice. The Wand tool was used to create a selection [region of interest (ROI)] delineating the white object.
2) For the quantification of FA crystals, the aim was to generate binary images where only FA crystals are white with a high degree of sensitivity and specificity (e.g., excluding artifacts and red blood cells). Color-thresholding of each full-scale image was done by using a narrow band-pass filter in the hue map (min = 25; max = 50) before recomposing with the original saturation and brightness maps. The resulting image was denoised using an iterated Despeckle command followed by an Analyze Particles command. The ROI that was established in the preprocessing step was then rescaled to the original dimension and restored, and raw integrated density was measured within the ROI.
3) For the quantification of the total tissue area, the aim was to generate binary images where all continuous renal tissue and tubular lumens are white (to exclude AKI-induced tubular dilatation effects) with a high degree of specificity. Each image was first downscaled by a factor of four by binning with averaging to simplify subsequent operations. Color-thresholding of the image was done using a wide band-pass filter in the red channel (min = 0; max = 155) and then recomposed with the original green and blue channels. The resulting image was denoised using the Remove Outliers command, processed using light Gaussian Blurring and Variance Filtering, and re-binarized by thresholding (min = 254; max = 255). The rescaled ROI established in the pre-processing step was then restored, and Raw Integrated Density was measured within the ROI. FA crystal content was computed by dividing the Raw Integrated Densities of each FA crystal image by that of the corresponding total tissue image. The image analysis pipeline underlying the FA crystal quantification is schematically shown in Fig. 2.

### FA Crystal Spatial Distribution Quantification

**Figure 3.**
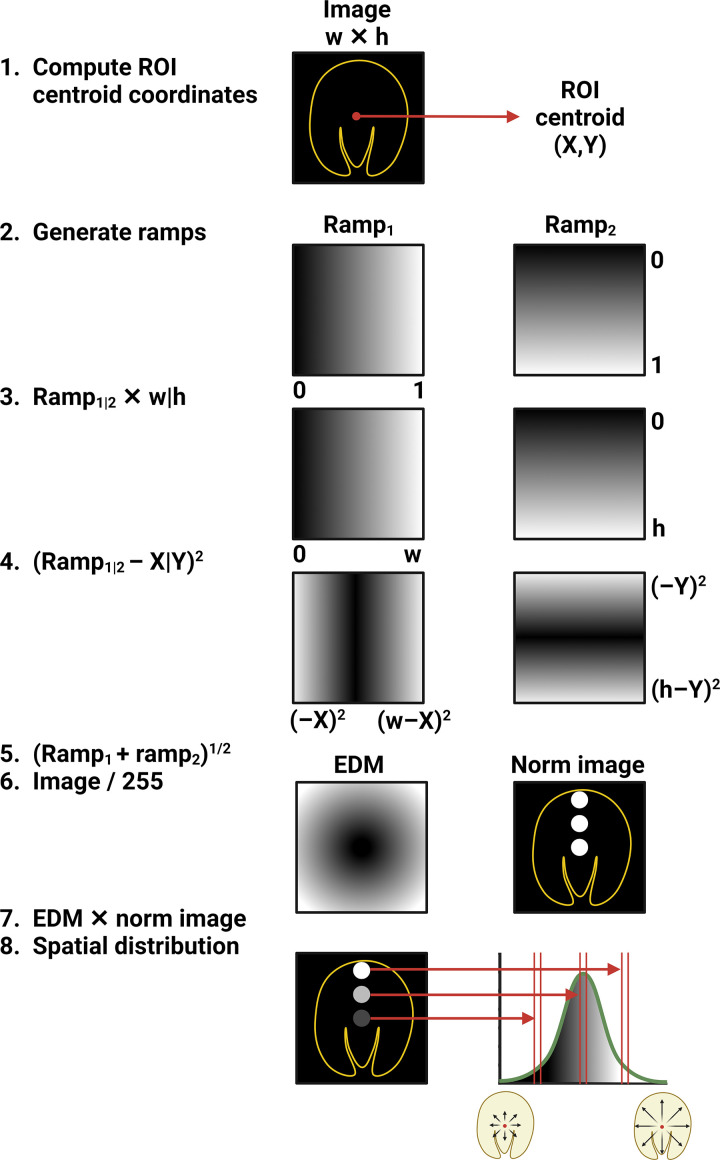
Workflow for the quantification of the folic acid (FA) crystal spatial distribution in segmented images after FA-induced acute kidney injury (FA-AKI). *1*: The centroid of the region of interest (ROI) delineating the slice was first computed. *2–6*: Vertical and horizontal ramp images were then generated and processed with image arithmetic following the Euclidean distance formula. *7 and 8*: Segmented binary crystal images generated previously were then applied as a mask onto the Euclidean distance map to render images where pixels corresponding to FA crystals have grayscale values equivalent to their distance relative to the ROI centroid. A histogram was then plotted to visualize FA crystal pixel count as a function of distance from the slice center. Created with BioRender.com.

FA crystal spatial distribution was conducted using a macro in FIJI software version 1.53t (code deposited and freely available at https://gitfront.io/r/AKMHamid/RWpkdKCDZT4J/FACrystalQuantMacro/). The centroid of the pre-established ROI in each image was first computed - the average position of all pixels within the ROI. Then, for each segmented FA crystal image, two 32-bit horizontal and vertical ramp images of equal resolution were created. Using image arithmetic commands, the Euclidean distance formula was then applied: the abscissa and ordinate values of the ROI centroid were subtracted from each pixel of the horizontal and vertical ramp images, respectively. The resultant images were then squared and added to each other using the Image Calculator function, and then the square root of the output image was computed producing a global Euclidean distance map (EDM). A 32-bit FA crystal EDM was then generated by first normalizing (dividing by 255) the previously computed 8-bit binary FA crystal image and then multiplying the output by the 32-bit global EDM such that each non-crystal pixel has a value of zero while that of every crystal pixel is equivalent to its distance from the ROI centroid. Finally, the ROI was restored in the FA crystal EDM, and a histogram with a bin width of 250 was compiled; distance in pixels was then manually converted to physical distance in accordance with the image scale. The digital workflow for the quantification of the FA crystal spatial distribution is schematically shown in Fig. 3.

### Raman Spectroscopy

Raman spectroscopy was performed using the Horiba LabRAM HR Evolution with the following setup: laser (785 nm; source power: 300 mW), grating (grooves density: 300 lines/mm), objective lens (FA purified crystal measurement: Olympus MPlanN, NA = 0.9; tissue sample measurement: Olympus UPLSAPO60XW, NA = 1.2), and detector (Horiba Synapse CCD). The grating was calibrated by reference Silicon peak using the laser, and optical filters (edge filter and holographic notch filter) were placed in the Raman beam path to selectively block the laser line (Rayleigh scattering) while allowing the Raman scattered light through to the detector. The spectra were acquired in the Raman shift range 500–1,900 cm^−1^ with a filtering spike. The absence of sample damage was confirmed by visual inspection after imposing the laser. The postprocessing (background subtraction and de-noising) of Raman spectrum data was performed by MATLAB-based open-source software (https://github.com/harubang2/Raman_processor) (31).

### Data Analysis and Statistics

All data were analyzed using GraphPad Prism 9. All immunoassays were fitted using a 4-parameter logistic model. Statistical analysis of all experiments was conducted using two-way ANOVA; α = 0.05. Tukey’s test was utilized for post hoc analysis to correct for multiple comparisons. All data are presented as means ± standard deviation.

All data were also tested for heteroscedasticity using the non-parametric Spearman’s rank correlation test conducted on predicted *Y* values and absolute residuals. Given the robustness of ANOVA to the violation of the homoscedasticity assumption under the condition of sample size homogeneity across experimental groups, a Spearman’s ρ (*R*_s_) threshold of ±0.7 was a priori dictated to indicate heteroscedasticity when significant (α = 0.05) (32). The threshold was chosen based on the previously suggested correlation coefficient strength categorization (33). The Anderson–Darling, D’Agostino–Pearson omnibus, Shapiro–Wilk, and Kolmogorov–Smirnov tests were collectively utilized to assess whether the residuals were Gaussian. In cases of heteroscedasticity or non-normality, the data were first logarithmically (log_10_) transformed before conducting the parametric statistical tests.

## RESULTS

### Image Processing Pipeline Accurately Captured FA Crystals

**Figure 4.**
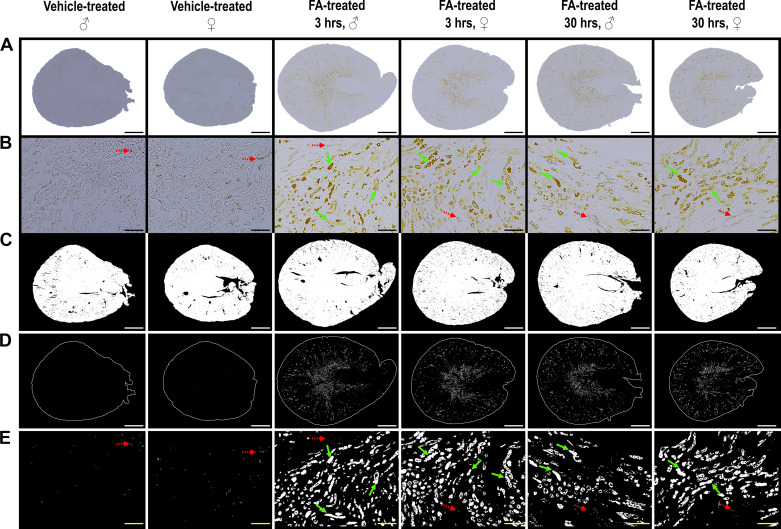
Representative images of transverse kidney sections and crystal quantification after folic acid (FA)-induced acute kidney injury (FA-AKI). Wild-type male (♂) and female (♀) C57BL6/JRj mice were intraperitoneally injected with FA or NaHCO_3_ (vehicle) and euthanized after 3 or 30 hours. *A*: representative stitched brightfield images of unstained transverse kidney sections. *B*: zoomed *insets* of the brightfield images (scale bar = 100 µm). *C*: processed binary total tissue images. *D*: segmented binary images of deposited FA crystals enclosed by a tissue-delineating perimeter. *E*: zoomed *insets* of the segmented binary images of deposited FA crystals corresponding to the same region as *B* (scale bar = 100 µm). Scale bar = 1 mm, unless otherwise indicated. Green solid arrows point to FA crystals while red dashed arrows point to red blood cells excluded from quantification.

FA treatment resulted in renal medullary and cortical deposition of FA crystals after 3 h, which were not cleared at the 30-h timepoint. Under bright-field microscopy, FA crystals presented as either yellow multiglobular or diffuse intratubular depositions predominantly concentrated in the medullary region (Fig. 4, *A* and *B*). Automated processing for tissue detection produced binary images accurately captured renal slices while excluding large anatomic (renal pelvis and vascular lumina) and artifactual voids (tears during cryosectioning) (Fig. 4*C*). Automated image segmentation sensitively isolated FA crystals without including red blood cells or artifacts such as undissolved OCT compound; processing yielded negligible false positive detections in the vehicle-treated mice (Fig. 4, *D* and *E*).

### Renal FA Crystal Content Is Sex Dependent in FA-AKI

**Figure 5.**
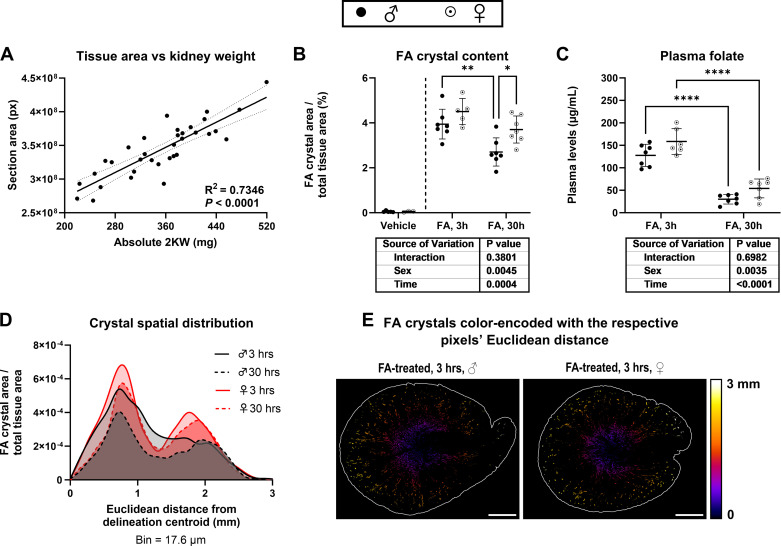
Folic acid (FA) renal crystal content and plasma concentration after FA-induced acute kidney injury (FA-AKI). Wild-type male (♂) and female (♀) C57BL6/JRj mice were intraperitoneally injected with FA or NaHCO_3_ (vehicle) and euthanized after 3 or 30 hours. *A*: simple linear regression analysis of digitally quantified kidney transverse section area and absolute two-kidney weight (2 KW). The dotted line indicates the 95% confidence interval. *B*: renal FA crystal content quantified as the area occupied by crystals as a percent of total tissue area in the section. *C*: plasma FA concentration. *D*: spatial distribution of FA crystals quantified as the pixel count in all deposited crystals at the indicated Euclidean distance relative to the centroid of the tissue-delineating perimeter. *E*: segmented grayscale images of deposited FA crystals color-encoded with the respective pixels’ Euclidean distance to the centroid of the shown tissue-delineating perimeter. Scale bar = 1 mm. Data are expressed as means ± SD, (*n* = 5 for male-vehicle; *n* = 3 or 4 for female-vehicle; *n* = 7 for male-FA-3h; *n* = 5 for female-FA-3h; *n* = 7 for male-FA-30h; and *n* = 7 for female-FA-30h). *P < 0.05, **P < 0.01, and ****P < 0.0001 between indicated groups by two-way analysis of variance with Tukey’s post hoc unless otherwise indicated (vehicle groups were not included in the statistical analysis).

Before quantifying FA crystal content as a percent of total tissue area, the total tissue quantification approach was validated by confirming a strong correlation between section area and absolute two-kidney weight (2 KW) (Fig. 5*A*). After 3 h, FA treatment resulted in the renal deposition of crystals with no discernable difference between males and females. After 30 h, FA-treated males but not females displayed a reduction in FA crystal content (Fig. 5*B*). Similarly, plasma folate was reduced after 30 h in both sexes, but females displayed a group effect for increased levels (Fig. 5*C*). FA crystal spatial distribution analysis revealed a bimodal distribution peaking at 0.70–0.80 mm and 1.80–1.90 mm from the computed renal centroid, representing most likely part of the medullary and cortical region, respectively. The distribution was comparable across both sexes and timepoints (Fig. 5*D*). The computed centroids of the tissue-delineating perimeter coincided with the inner medulla and rendered Euclidean distance maps consistently relative to the central inner medulla (Fig. 5*E*).

**Figure 6.**
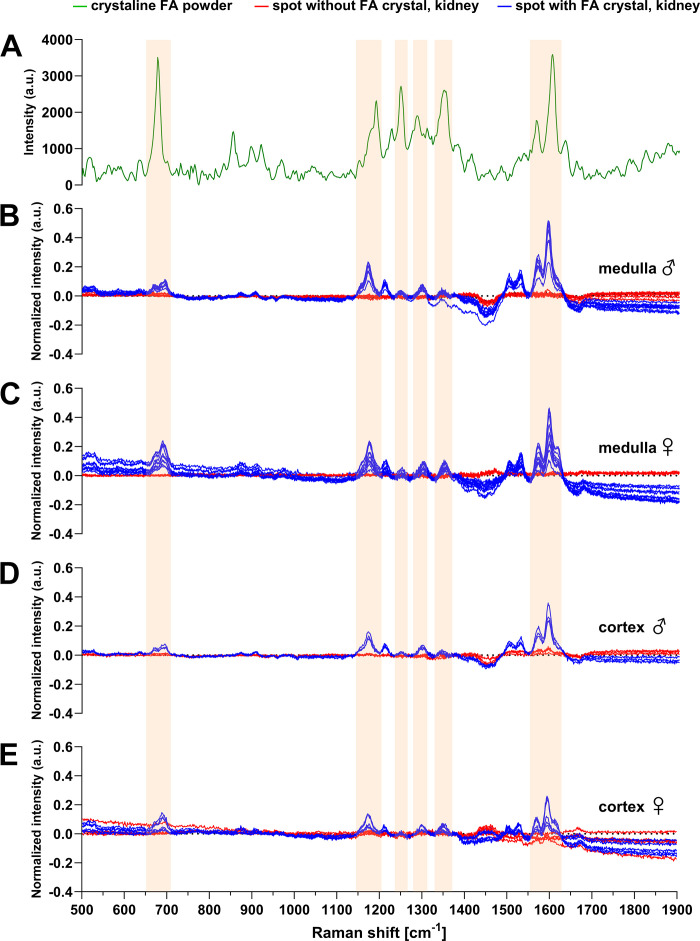
Raman shifts of folic acid (FA) crystalline powder and of representative transverse kidney sections after FA-induced acute kidney injury. Wild-type male (♂) and female (♀) C57BL6/JRj mice were intraperitoneally injected with FA or NaHCO_3_ (vehicle) and euthanized after 3 h. *A*: Raman shift of the crystalline FA powder. *B–E*: Ramen shift of spots without (red lines) and with (blue lines) FA crystals of an area in the medulla (*B* and *C*) and cortex (*D* and *E*) in a male (*B* and *D*) and female (*C* and *E*) kidney.

To confirm that the quantified crystals consist of FA, we took advantage of Raman spectroscopy. We confirmed the Raman shift of crystalline FA powder (34, 35) and compared it to cortical and medullary spots containing FA crystals versus spots containing no crystals. The Raman signal obtained from FA crystals in the kidney was largely preserved when compared to the Raman signal obtained from crystalline FA powder (Fig. 6). No signal was detected in spots containing no crystals (Fig. 6) and the vehicle-treated mice (not shown).

### A Subset of Kidney Injury and Inflammatory Markers Is Sex Dependent in FA-AKI

**Figure 7.**
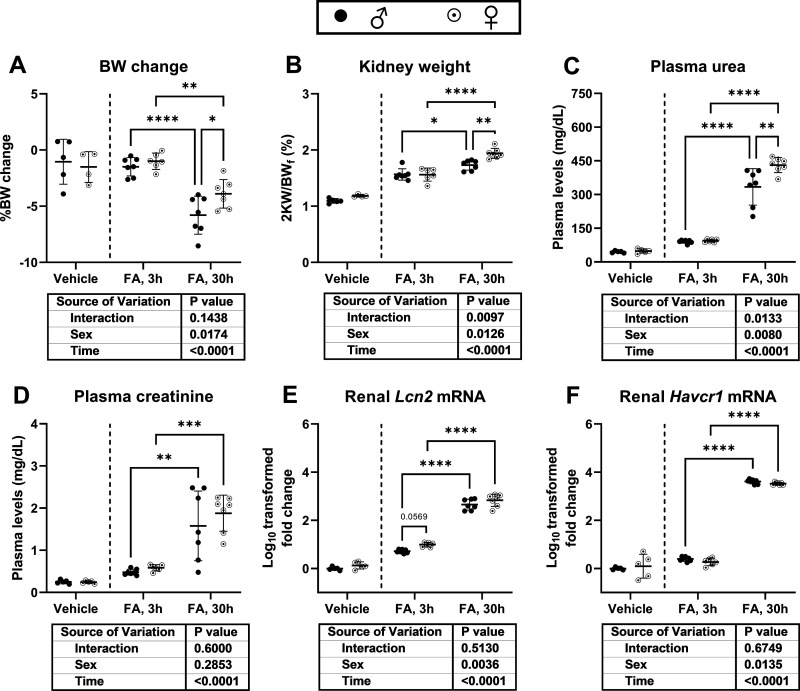
Kidney injury markers after folic acid (FA)-induced acute kidney injury (FA-AKI). Wild-type male (♂) and female (♀) C57BL6/JRj mice were intraperitoneally injected with FA or NaHCO_3_ (vehicle) and euthanized after 3 or 30 h. *A*: percent change in mouse body weight. *B*: percent two-kidney weight per body weight (2 KW/BW). *C* and *D*: plasma urea and creatinine levels. *E* and *F*: renal *Lcn2* mRNA [neutrophil gelatinase-associated lipocalin (NGAL)] and *Havcr1* mRNA [kidney injury molecule-1 (KIM-1)] expression fold change. Data are expressed as means ± SD, (*n* = 5 for male-vehicle; *n* = 4 or 5 for female-vehicle; *n* = 7 for male-FA-3h; *n* = 6 for female-FA-3h; and *n* = 7 for male-FA-30h; and *n* = 7 for female-FA-30h). *P < 0.05, **P < 0.01, ***P < 0.001, and ****P < 0.0001 between indicated groups by 2-way analysis of variance with Tukey’s post hoc (vehicle groups were not included in the statistical analysis).

Whereas, after 30 h of FA treatment, females suffered a reduced relative loss of BW than males, they exhibited higher 2 KW/BW ratios and plasma urea, which is in line with their higher renal FA crystal content (Fig. 7, *A*–*C*). However, no sex-dependent differences were observed in plasma creatinine at either timepoint (Fig. 7*D*). *Lcn2* [neutrophil gelatinase-associated lipocalin (NGAL)] and *Havcr1* [kidney injury molecule-1 (KIM-1)] mRNA was upregulated upon injury and increased over time (Fig. 7, *E* and *F*). *Lcn2* mRNA expression displayed a significant sex-group effect with higher expression in female mice, whereas *Havcr1* mRNA expression displayed a significant sex-group effect with higher expression in male mice.

**Figure 8.**
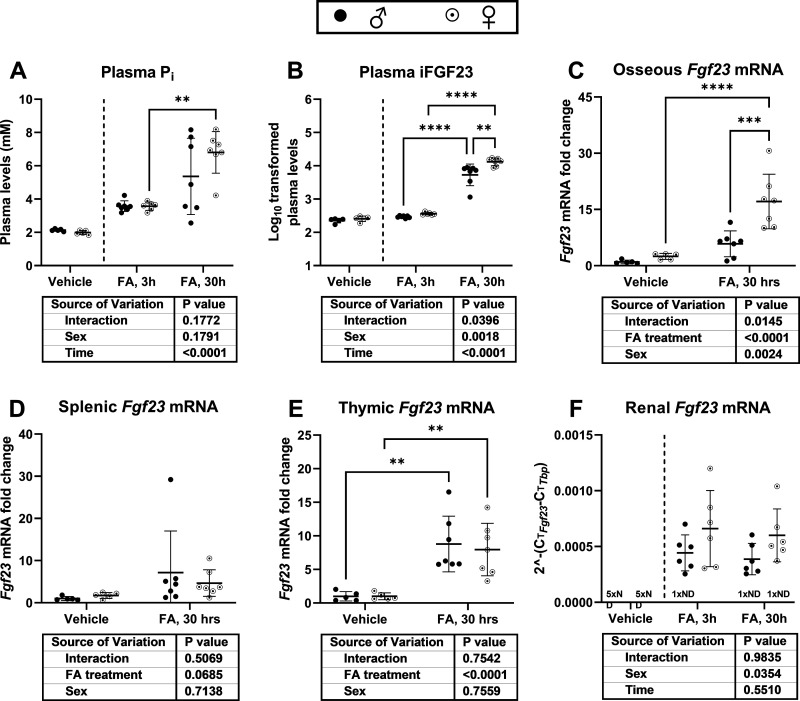
Plasma P_i_ and plasma and tissue mRNA expression of fibroblast growth factor 23 (FGF23) after folic acid (FA)-induced acute kidney injury (FA-AKI). Wild-type male (♂) and female (♀) C57BL6/JRj mice were intraperitoneally injected with FA or NaHCO_3_ (vehicle) and euthanized after 3 or 30 h. *A* and *B*: plasma P_i_ and log-transformed plasma intact (i)FGF23 (native units: pg/mL). *C*–*E*: osseous, splenic, and thymic mRNA expression fold change at 30 h after FA treatment. *F*: renal *Fgf23* mRNA expression after 3 h and 30 h of FA treatment. ND = non-detectable. Data are expressed as means ± SD, (*n* = 5 for male-vehicle; *n* = 5 for female-vehicle; *n* = 6 for male-FA-3h; *n* = 6 for female-FA-3h; *n* = 6 for male-FA-30h; and *n* = 6 for female-FA-30h). *P < 0.05, **P < 0.01, ***P < 0.001, and ****P < 0.0001 between indicated groups by two-way analysis of variance with Tukey’s post hoc (vehicle groups were not included in the statistical analysis of *A*, *B*, and *F*).

**Figure 9.**
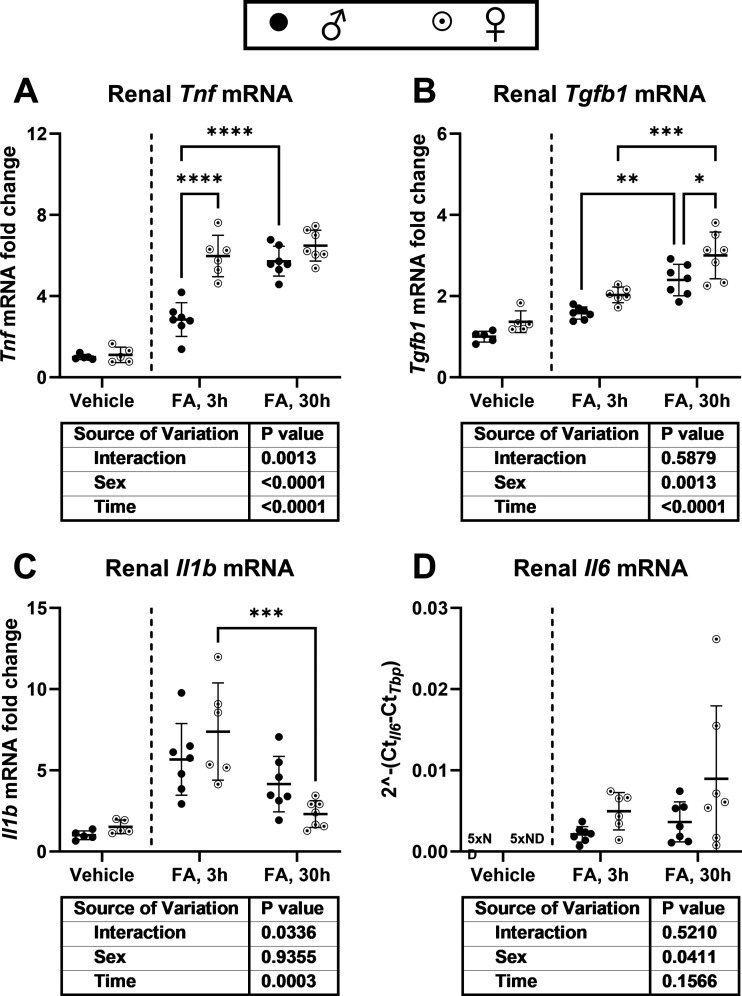
Renal inflammatory cytokine mRNA expression after folic acid (FA)-induced acute kidney injury (FA-AKI). Wild-type male (♂) and female (♀) C57BL6/JRj mice were intraperitoneally injected with FA or NaHCO_3_ (vehicle) and euthanized after 3 or 30 hours. *A–D*: renal *Tnf*, *Tgfβ1*, *Il1β*, and *Il6* mRNA expression fold change. ND = non-detectable. Data are expressed as means ± SD (*n* = 5 for male-vehicle; *n* = 5 for female-vehicle; *n* = 7 for male-FA-3h; *n* = 6 for female-FA-3h; *n* = 7 for male-FA-30h; and *n* = 7 for female-FA-30h). **P < 0.01, ***P < 0.001, and ****P < 0.0001 between indicated groups by two-way analysis of variance with Tukey’s post hoc (vehicle groups were not included in the statistical analysis).

Plasma P_i_ increased in mice with FA-AKI over time in males and females. Furthermore, females exhibited over twice the plasma level of iFGF23 of males at the 30-h timepoint (Fig. 8, *A* and *B*). This was accompanied by a substantially greater upregulation of osseous but not splenic or thymic *Fgf23* mRNA. In the kidney, *Fgf23* transcriptional induction was detected in all FA-treated groups, with a significant sex-group effect (Fig. 8, *C*–*F*). Furthermore, upon FA treatment, renal *Tnf* (tumor necrosis factor, 3-h timepoint) and *Tgfβ1* (transforming growth factor β1, 30-h timepoint) mRNA was further upregulated in females than in males. No sex-dependent difference in renal *Il1β* (interleukin-1β) mRNA was detected, whereas renal *Il6* (interleukin-6) mRNA exhibited a sex-group effect (Fig. 9, *A*–*D*).

## DISCUSSION

In this study, we describe a novel and simple method for the fixation and preparation of kidney sections toward the visualization and quantification of FA crystals in the mouse FA-AKI experimental model. We demonstrated the utility of this method by characterizing the model in terms of sex-dependence over two timepoints and showed that FA crystals deposit in a bimodal spatial distribution in the kidney, with a higher crystal content in females, and demonstrated a concomitant effect on a subset of kidney injury and inflammatory markers.

FA-AKI is an increasingly utilized experimental model in rodents; however, a remarkable paucity of information regarding the model’s pathomechanism and sex dependence has generated the impetus for the development of a tool for the quantification of the root pathological culprit: renal FA crystals (10). A major obstacle in the visualization of FA crystals lies in their high propensity to elution in typical histological practice on account of the pH-dependent solubility of FA (36, 37). In our tissue fixation and preparation protocol we circumvented this issue by strictly adhering to the use of an acidic fixative solution, entirely avoiding the use of buffered and alkaline solutions (e.g., PBS and alkaline mounting media), and minimizing the duration of steps in non-acidic solutions and organic solvents. As opposed to the previously described tissue fixation protocols for FA crystal visualization using absolute ethanol, Carnoy’s solution (ethanol-chloroform-glacial acetic acid), and AAF solution (ethanol-formalin-glacial acetic acid), formal saline fixation results in a lesser degree of tissue shrinkage, over-hardening and consequent crumbling, and distortion, which would otherwise hamper the possibility of obtaining high-quality whole-slice images and interfere with downstream quantification (22, 25, 38). Previously used approaches also included electron microscopy, which greatly restricts observational breadth; snap-freezing without fixation, which affects sample stability under prolonged imaging; and homogenate spectrophotometry, which wholly eliminates spatial insight (12, 14, 39). Therefore, together with the demonstrated possibility of unbiased automated imaging, the histological approach described herein is straightforward and tissue-preserving.

The image analysis pipeline outlined in this study incorporated both the RGB and HSB color spaces. The commonly used RGB thresholding was applied for the segmentation of the total tissue, whereas the generation of a tissue-delineating perimeter and the segmentation of the FA crystals was accomplished using HSB thresholding. Thresholding images in the hue channel of the HSB color space proved advantageous as it yielded highly sensitive detection of FA crystals, owing to their distinctive hue, without incorporating saturation information that would otherwise pose a challenge in RGB thresholding. For example, by thresholding in the hue channel, we circumvented the impact of undissolved and superimposed OCT on the image saturation and brightness. False positive detections were limited to red blood cells, which were reliably excluded given their contrasting morphology. The sensitivity, selectivity, and robustness of this fully automated quantification pipeline further add to the advantages of this method.

To confirm the applicability of the method described herein, we showed that the FA-AKI model is underlain by a bimodal spatial distribution of renal FA crystal deposition and bears a stark sex dependence. The demonstrated bimodal spatial distribution, in the medullary and cortical regions, may be a result of the pH-dependent solubility of FA and the changing pH along the nephron (36, 40, 41). Thus, the first crystallization peak in the medulla could be caused by the acidification of the urine in the collecting duct, whereas the second, less pronounced peak may originate from the initial drop of pH in the proximal tubule. In addition, we showed that renal FA crystal content is higher in females at the 30 h timepoint, which either highlights artifactual anatomical sexual dimorphism (disproportional FA dosing given its normalization to BW) or suggests a difference in crystal deposition propensity or clearance. Male rodents have higher inulin clearance rate and urinary output compared to female rodents which may promote the clearance of FA crystals and thereby mitigate renal injury (42–44). This may contribute to a faster clearance of FA crystals in males and concomitant lower renal injury. Concurrently, females displayed a higher 2 KW/BW ratio, plasma urea and iFGF23, and renal inflammatory cytokine expression suggesting worse kidney injury and function in female mice (45–47). Plasma creatinine upon FA-AKI was similar in both sexes but might have been influenced by the fact that male mice have more muscle mass than females (48), which can lead to comparable increases in plasma creatinine levels despite lower injury (49–51). Furthermore, the expression of the injury markers *Lcn2* and *Havcr1* was increased with increasing injury over time; however, we were not able to discern the difference in injury between male and female mice with the gene expression of these two injury markers. The fine-tuning of their regulation might be multifactorial and not solely representative of kidney injury. Taken together, the observed divergence in parameters pertaining to renal injury thereby warrants cautious data interpretation in mixed-sex studies utilizing the FA-AKI model.

Plasma P_i_ is an important determinant of FGF23 and a rise in plasma P_i_ due to high dietary P_i_ intake or renal injury is paralleled by higher plasma FGF23 (52, 53). Indeed, the rise in plasma P_i_ in FA-AKI was accompanied by a rise in FGF23; however, despite a similar increase in plasma P_i_, the rise in plasma FGF23 and osseous *Fgf23* expression upon FA-AKI was twice as high in female compared with male mice. Recently, it was shown that the regulation of FGF23 is multifactorial and includes, in addition to P_i_, proinflammatory cytokines and factors of iron and energy metabolism (54–58). Worse kidney function and higher expression of proinflammatory cytokines may be responsible for the higher FGF23 expression in female compared with male mice upon FA-AKI.

A limitation of this method is the lack of optimization for additional tissue staining procedures. Histological staining or immunohistochemistry would enable FA crystal localization with higher specificity and the characterization of the model in terms of nephrotoxic mechanism; however, such additions to the protocol would necessitate thorough optimization and adherence to acidic environments. Our experiments demonstrated that FA crystals can also be reliably visualized by polarized microscopy (unpublished data), which may be a preferable approach for downstream quantification in the case of stained tissue, albeit with a requisite modification of the image analysis pipeline towards saturation, in lieu of hue, channel thresholding for crystal segmentation. We have also found that VectaMount is an optimal mounting medium for crystal preservation, particularly when compared to alkalinized mounting media, but this medium may be incompatible with immunofluorescence staining.

Another limitation of the histological approach is the omission of PBS washing, which would ordinarily dissolve the OCT compound precluding its interference by folding onto the slice. Although this artifact has no bearing on the approach implemented herein, it may affect the quantification of stains potentially incorporated in the protocol.

The FA crystal spatial distribution analysis approach adopted in this method portrays the total area occupied by crystals as a function of a distance range (histogram bins) from the computed renal centroid to provide the proportion of total crystals in a given anatomical region of the kidney. However, this distribution does not reflect FA crystal density at a given radius from the computed renal centroid: this can be accomplished by dividing the total crystal area in a given distance range by the bin’s corresponding ring-shaped area, which can be calculated from the radii of the circles bounding the range.

In conclusion, this method constitutes an important tool for studies involving the FA-AKI experimental model as it can serve as a valuable control in excluding a confounding effect of early or prophylactic interventions on the initial renal insult of FA crystal deposition. For instance, as FA solubility is steeply pH-dependent, it is conceivable that experimental interventions impacting urine pH may alter FA crystal deposition. FA crystal visualization and quantification may also prove valuable for studies aiming to characterize the pathomechanism of the FA-AKI model.

## DATA AVAILABILITY

Both FIJI batch macFros for the quantification of FA crystal content and spatial distribution as well as the detailed ingredient list of the mouse chow used in the experiments herein can be found at https://gitfront.io/r/AKMHamid/RWpkdKCDZT4J/FACrystalQuantMacro/. All raw microscopy images generated in the study can be provided upon reasonable request.

## GRANTS

This work was supported by grants from the Swiss National Center for Competence in Research NCCR Kidney.CH (to C.A.W. and D.E.-S.), the Olga Mayenfisch Foundation (to D.E.-S.), and the Gottfried und Julia Bangerter Rhyner Foundation (to D.E.-S).

## DISCLOSURES

No conflicts of interest, financial or otherwise, are declared by the authors.

## AUTHOR CONTRIBUTIONS

A.H., C.A.W., and D.E.-S. conceived and designed research; A.H., E.M.P.A., S.S.L., and D.E.-S. performed experiments; A.H. and S.S.L. analyzed data; A.H. and D.E.-S. interpreted results of experiments; A.H. prepared figures; A.H. drafted manuscript; A.H., S.S.L., C.A.W., and D.E.-S. edited and revised manuscript; A.H., E.M.P.A., S.S.L., C.A.W., and D.E.-S. approved final version of manuscript.
